# Quarantinable Diseases

**Published:** 1918

**Authors:** S. B. Grubbs

**Affiliations:** Newport News, Va.; Surgeon, U. S. Public Health Service


### QUARANTINABLE DISEASES.

**BY S. B. Grubbs, M.D., Newport News, Va.**
**Surgeon, U. S. Public Health Service**

All eyes are now focused on shipping, and Hampton Roads, as a port, is rapidly increasing in importance. I thought, therefore, it would be approapriate to say something about the serious epidemic diseases and the methods of modern quarantine to exclude them from this country. These diseases, like the fighting in the present war, may seem a long way off, and that separated from they by a protecting ocean we have little to fear, but I can assure you that the danger is very real, and that the greatest vigilance is necessary.

During the last session, Congress appropriated half a million dollars for increased. quarantine facilities on the Atlantic Coast, and of this $223,000 is for Hampton Roads. $143,000 is for Construction on Craney Island, where plans call for six new barracks, a hospital, a sterilizing building, a power house, office and surgeon's quarters, in addition to those now there. This will allow the detention of 1,490 passengers at one time. The balance of the appropriation will be used for equipment and fumigating machinery.

The United States quarantine regulations mention six diseases which, on account of the terrible ravages they have made in the past, must be excluded from our shores at all cost. These diseases are plague, cholera, typhus fever, yellow fever, smallpox and leprosy. Leprosy is a chronic affection and smallpox is always present here and there in this country, on account of our laxity in vaccination. Plague, cholera, typhus fever and yellow fever, however, are diseases with awful records of death and misery that must be held away if possible and throttled promptly if discovered within our territory.

It is interesting to go back twenty years and compare our efforts and our methods against these diseases then and now, especially so with yellow fever which has until recent years menaced the lives and prosperity of the Southern States. Twenty years ago yellow fever was feared above all other diseases. Towns were abandoned, trade was interrupted and panic followed even the rumors of its presence. Quarantine restrictions against yellow fever twenty years ago were strict, but in spite of them the disease would from time to time gain access to our Southern ports and spread steadily until frozen out in the fall. This was not always due to lack of zeal or to carelessness, as I can testify.

I was in Havana when we took possession of that ctiy on January 1, 1899. Havana had for years been one of the pest holes of the world and one of the first tasks before us was to get rid of yellow jack. A general clean-up of the city was carried out in a most thorough manner, regardless of expense or protests. Houses, sidewalks, and streets were all alike scrubbed, scoured and disinfected, until at times the smell of chlorine suggested stories of a modern gas attack. Yet, after all that, I saw over fifty cases of yellow fever at one time and during 1899 and 1900 there were nearly four hundred deaths occurring in Havana, the clean and spotless. While this was going on, the quarantine service, in its conscientious efforts to protect the Southern States, was disinfecting every ship, every piece of baggage and even every letter and parcel leaving the island. In twenty-four hours preceding the sailing of the Saturday steamer, several large lighter loads of trunks and hand baggage had to be each unpacked, the contents sterilized by steam, and repacked. Those who are appalled at the prospect of packing one trunk may wonder how we did this. It was a difficult task, but I may explain that all disinfected baggage was delivered on board the steamer with orders that nothing should be opened until well out at sea. This was to prevent the supposedly pestilential air from entering the now germ-free trunks, but it also served the very real purpose of saving us from the vengeance of the owners, for, while we were as careful as possible, our men could not always repack as skillfully as a lady's maid.

All this laborious and expensive work on land and sea seemed to have little effect upon yellow fever, because the time, effort and money were expended on cleaning and general sanitation instead of being concentrated on the weakest point of the particular disease in question.

As soou as all efforts were directed against the mosquito and nothing else, the disease stopped, not only in Havana, but elsewhere, and the Central American coast changed from poverty and misery to opulence and beauty.

This is a good example of the necessity of finding the weak link in the connecting chain between the infecting person and the person infected by him and concentrating our strength to break that link.

Clean or dirty, beautiful or ugly, matters little in stopping disease; it is the line of disease communication that must be cut. This applies to the common communicable diseases in our cities as well as to yellow fever and plague, and money appropriated for the prevention of disease should nt be wasted for cleaning, however desirable that may be.

Each of the so-called quarantinable diseases has a weak spot where we concentrate our efforts. With cholera it is the human carrier; with yellow fever, typhus fever plague it is the insect carrier; for these three most destructive of all epidemic diseases are dependent absolutely each upon a different insect to carry its germs from the sick to the well.

Cholera, like typhoid fever, is an intestinal disease and is spread by infected body discharges. As the symptoms of cholera are severe, a patient on an arriving vessel is easily discovered and may be isolated from those he may infect. Others, however, who are apparently well, may carry the germs and give the disease to those about them. "That the healthy human being may for months or years carry organisms which work disaster to his more susceptible neighbor has long since been confirmed," not only for cholera but for other diseases. These co-called "carriers" of cholera are the greatest dangers, as they show no signs by which they may be detected unless we call to our aid the bacteriological laboratory and the microscope. Where cholera has occurred on a vessel or the passengers come from a cholera infected district, all hands must be examined in order thta no carrier may pass, to spread the disease to those on shore. Every well equipped quarantine station, therefore, must have a laboratory for cholera examinations, and the personnel of such station must be trained in the special methods by which a thousand or more of such examinations may be made within twenty-four hours.

Yellow fever is transmitted by one species of mosquito, the Aedes calopus, and modern quarantine endeavors to discover the sick, destroy any mosquitoes on shipboard, and hold susceptible persons six days, as symptons of this disease will appear in that time or not at all. Fortunately, mosquitoes are quickly affected by certain gases, especially sulphur dioxide or hydrocyanic acid gas.

The present day yellow fever quarantine does not delay shipping unduly or annoy travel, but it is reasonably effective. If the disease ever again invades this country it will meet a quick defeat. It may be confidently asserted that the problem of yellow fever has been solved, and we may hope with Carter that the disease may be made to disappear entirely and forever.

Typhus fever and bubonic plague are aristocrats amongst the scourages of mankind, if we are to judge them by the length of their record, by the history they have made, and by the slaughter they have caused. In all the ages during which they have left their cruel marks upon mankind, no intelligent or effective defense has ever been carried out against them until the twentieth century, and the distinction between the two diseases has been confused down to comparatively modern times, even by medical writers. This was natural, for not only is there a resemblance in the symptoms which prevented most ancient writers from clearly differentiating the two, but they have frequently occurred in one and the same epidemic. We understand this now, as both diseases are transmitted by vermin, the body louse being the immediate agent in the propogation of typhus fever and the rat flea in the propagation of the other.

Typhus fever was distinguished as a distinct disease by Frascatorius as early as 1546, but it was not so generally recognized in Europe until the eighteenth century. It is entirely different as to symptoms and method of spread from typhoid fever, which unfortunately was named from its supposed resemblance to the older and then better known disease. Typhus was doubtless the epidemic disease described by Thucydides. It was probably the plague mentioned by Plutarch as causing the death of Pericles, and it can be traced continuously through lay and medical literature. "During the thirty years' war the whole of Central Europe was devasted by famine and typhus. They were rempant in the wake of Napoleon's armies, and thousands perished by typhus in the retreat from Moscow. In the Crimea typhus decimated the ill-fed army of the French," and during our Civil War the disease was common—at least in the prisons.

At present, despite modern hygiene, typhus fever has been widely spread in areas where invasion has been followed by famine and misery among the civil population, while revolution has greatly increased it in Mexico, in which country the disease has long existed.

The officers at our maritime quarantine stations must be constantly on the alert for typhus fever. Besides careful inspection to detect sickness, it may be necessary, even when all are well, to clean the persons and clothing of all passengers from infected districts in order to make them lice free. To do this, it is necessary to handle rapidly large numbers of ignorant and suspicious persons in whose lives bathing has been seldom considered necessary. Special apparatus is necessary to destroy vermin in clothing and baggage. For this purpose we have the choice of steam, dry heat and the lethal gases. The steam and dry heat methods require unpacking and repacking of baggage. Steam leaves the clothes damp; dry heat causes injury. Of the gases, hydrocyanic acid gas is ideal as it is selective in action, destroying all animal life wtihout injury to any material. The thing needed is to get the gas in contact with the insect that may be in the centre of a bag of clothers. Working on this problem two years ago I decided to try a process recommended by the Department of Agriculture for the destruction of boll worms in imported cotton bales. By this method a vacuum is created in the metal chamber holding the baggage, after which cyanide gas is admitted and then air, until atmospheric pressure is restored. The gas is thus forced into the very center of every package, killing any animal life therein without so much as lifting a lid or opening a bundle. This process is now in operation at the Boston Quarantine Station and will be installed in the up-to-date sterilizing building planned for Craney Island. I believe it will be of service in many other ways against vermin and insect pests.

Bubonic plague is contracted from plague-infected rats and is probably the most serious of pestilential diseases. It was constantly referred to by the ancient historians and poets. The epidemics referred to by Homer and in the Old Testament as well as those of a later date which almost depopulated Asia, were probably plague. This disease, known by the dread name of "The Black Death," ravaged Europe during the middle ages, the most notable periods being the epidemic that spread over Europe in the years of 1348 and 1349 and the great plague of London, in 1665. The epidemic of 1348 killed a large percentage of the population of Europe and according to Gasquet brought about an economic revolution, the like of which has never been caused except by war or by a social upheaval such as the French Revolution. Italy is dotted with shrines and churches erected in supplication to St. Sebastian and St. Roch, the protectors of the people against this disease, and art of that period has used plague repeatedly as a subject.

Plague apparently advances and recedes in waves so that during part of the nineteenth century it had receded, possibly existing nowhere except in India or China, among the marmots, which are small fur-bearing rodents. But we are now in the midst of another pandemic. Happily we are in possession of accurate information on the epidemiology of the disease, so that in advanced countries at least no great loss of life should take place, but it is to be regretted that more advantage is not being taken of our new knowledge. Every city and especially every seaport should allow nothing but rat-proof construction. Thus we would prepare in advance instead of waiting until the disease appears and in the midst of alarms be forced to do hurriedly and at great cost what we may now do at leisure and at slight expense.

Plague is advancing upon us both by the Atlantic and the Pacific. The Hawaiian Islands, San Francisco, Seattle, several Pacific ports of Mexico and of South America have been infected. By way of the Atlantic, Portugal, the Canary and Azores Islands, Rio, Bahia, Rosario and other South American ports have suffered. English and Scotch ports have reported cases and, what seems nearer home, Porto Rico, Cuba and New Orleans.

Plague has usually reached this country from ports where the infection was not recognized or, if known, was denied. Even when its presence is promptly acknowledged, the measures that may be taken to prevent infected rats from crossing the seas are none too satisfactory. Theoretically it is simple, namely, killing the rats on each ship arriving, but practically it is very difficult. Rats hidden away in the many corners of the vessels or in the cargo are reached with great difficulty and the cargo may be injured by fumigation or delays. In the modern fumigation of vessels we rely upon two gases, namely, sulphur dioxide, which is generated by burning sulphur, and hydrocyanic acid gas, sometimes called Prussic acid gas, which is generated from the combination of sodium cyanide and dilute acid. Fumigation of a ship by sulphur dioxide requires from twelve to twenty-four hours. With cyanide it can be done in four to six hours. Sulphur dioxide is injurious to much that a ship contains, while cyanide injures nothing but animal life. On the other hand, cyanide is so deadly that it must be used with the greatest care.

To sum up, we know definitely the ways in which four of the so-called quarantinable diseases are spread. Yellow fever, typhus fever and plague are carried by insects and cholera by human beings. We must not let these carriers of disease enter our ports and we must not unduly delay shipping. To accomplish this requires a trained personnel and ezpensive equipment. That it is worth the effort and expense there can be no doubt, as these diseases are so terrible in their toll of life and cost to commerce that everything possible must be done to keep them away.—Virginia Medical Monthly.

